# The complete mitochondrial genome of the wild silkmoth *Antheraea yamamai* from Heilongjiang, China (Lepidoptera: Saturniidae)

**DOI:** 10.1080/23802359.2021.1945975

**Published:** 2021-07-14

**Authors:** Shu-Wei Sun, Jing-Chao Huang, Yan-Qun Liu

**Affiliations:** a College of Agronomy, Eastern Liaoning University, Dandong, China; b College of Bioscience and Biotechnology, Shenyang Agricultural University, Shenyang, China

**Keywords:** *Antheraea yamamai*, mitochondrial genome, phylogenetic relationship, intraspecific variation

## Abstract

Here, we reported the complete mitochondrial genome of *Antheraea yamamai* Guérin-Méneville (1861) collected in Heilongjiang Province, China. The mitochondrial genome is 15,341 bp and encodes 13 protein-coding genes, two ribosomal RNA genes, and 22 transfer RNA genes. Sequence comparison identified 22 SNVs in the *A. yamamai* mitochondrial genomes between Chinese and Korean populations, indicating a low intraspecific variation between the two populations . Phylogenetic analyses with maximum-likelihood and Bayesian inference methods revealed a close relationship between *A. yamamai* and *Antheraea frithi* and supported the relationship among *Antheraea* species (((*A. yamamai* + *A. frithi*) + *A. pernyi*) + *A. assamensis*).

The wild silkmoth *Antheraea yamamai* Guérin-Méneville (1861) is one of the most well-known wild silkmoths belonging to the family Saturniidae. This species is distributed throughout China, Korea, Japan, and Russia. *Antheraea yamamai* produces green silk called Tensan silk, which has been considered a new biomaterial and a useful product in human health research and economic fields (Kim et al. 2018). The pupae of this species are also considered to be potential insect-derived food resources with high nutritional value (Yue et al. 2017). To date, this insect is still a noncultivated silkmoth reared outdoors. To protect this economically important silkmoth resource as well as its living environment, the province-level Shuguang Nature Reserve (N45°47′40′′ ∼ 45°35′27′′; E131°03′20′′ ∼ 131°11′17′′) was created in Heilongjiang Province, China (Hou et al. 2010). In the present study, we reported the complete mitochondrial genome of this wild silkmoth collected in the Shuguang Nature Reserve to provide basic genetic information.

The *A. yamamai* eggs collected in the Shuguang Nature Reserve were kindly provided by Tian-Mao Wang, Developmental Center of Heilongjiang Provincial Sericulture and Bee Industry (N45°54′; E126°39′), Harbin, China. After hatching, the larvae were fed with the leaves of *Salix viminalis* in the Silkmoth Experimental Field of Shenyang Agricultural University (N41°50′1.08′′; E123°34′21.92′′). An adult specimen was deposited at the Department of Sericulture, Shenyang Agricultural University, China (https://www.syau.edu.cn/, Dr. Yan-Qun Liu, liuyanqun@syau.edu.cn) under voucher number SILKMOTH_YAMAMAI_01. A hind leg was used to extract the total genomic DNA, which was also deposited at the Department of Sericulture. Long PCR amplification was used to obtain the whole mitochondrial genome with two species-specific primer pairs. After purification by gel extraction, the amplification products were mixed equally with a sample from *Antheraea pernyi* Qing_6 and then sequenced on the Illumina PE 150 platform. The resulting clean reads were subjected to the Galaxy web server at usegalaxy.org (https://usegalaxy.eu/) to assemble the mitochondrial genome (Jalili et al. 2020).

The whole mitochondrial genome of *A. yamamai* presented here is 15,341 bp in length, thus exhibiting a highly similar size as the reference genome from the Korean population (15,338 bp; EU264055; Kim et al. 2009). This genome also encodes 37 mitochondrial genes, including 13 protein-coding genes, 2 ribosomal RNA genes, and 22 transfer RNA genes, showing an identical genomic component and gene order with known Saturniidae species. The length of the A + T-rich region for *A. yamamai* is 334 bp, which is similar to those of *Antheraea assamensis* and *Antheraea frithi* (328 ∼ 334 bp) but much shorter than that of *A. pernyi* (516 ∼ 552 bp). Further analysis revealed that the presence of a 38 bp tandem repeat unit (Arunkumar et al. 2006) in *A. pernyi* resulted in the size variation of the A + T-rich region mentioned above, thus contributing to the length variation of whole mitochondrial genomes of the genus *Antheraea*.

By sequence comparison, we identified 22 SNVs (19 SNPs and three indels) between the two *A. yamamai* mitochondrial genomes. Three single-base deletion mutations occurred in the intergenic spacer region *ND4-ND4L*. Among these 19 SNPs, four were present in *rRNAs* and one was in the intergenic spacer region *ND2-tRNA^trp^*; the remaining 14 occurred in protein-coding genes, resulting in five amino acid changes. The number of SNVs between the two *A. yamamai* mitochondrial genomes were smaller than those between the cultivated and noncultivated *A. pernyi* mitochondrial genomes [264 SNVs (213 SNPs and 51 indels)] and between the two *A. assamensis* mitochondrial genomes [246 SNVs (158 SNPs and 88 indels)], but similar to those between strains of *A. pernyi* (Li et al. 2021). These results indicated a low intraspecific variation between Chinese and Korean populations of *A. yamamai*, suggesting that they might be derived from a common population.

For phylogenetic analysis (Figure 1), seven whole mitochondrial genomes from *Antheraea* species were included (Kim et al. 2009; Shantibala et al. 2017; Singh et al. 2017; Zhong et al. 2017). Six mitochondrial genomes from non-*Antheraea* species were also included. *Bombyx mori* (Lu et al. 2002) served as an outgroup. A maximum-likelihood tree was constructed with IQ-TREE 1.6.12 (Nguyen et al. 2015), and a Bayesian inference tree was constructed with Mrbayes v.3.2 (Ronquist et al. 2012). Our phylogenetic analyses revealed a close relationship between *A. yamamai* and *A. frithi* and supported the relationship of the genus *Antheraea* (((*A. yamamai* + *A. frithi*) + *A. pernyi*) + *A. assamensis*).

**Figure 1.**
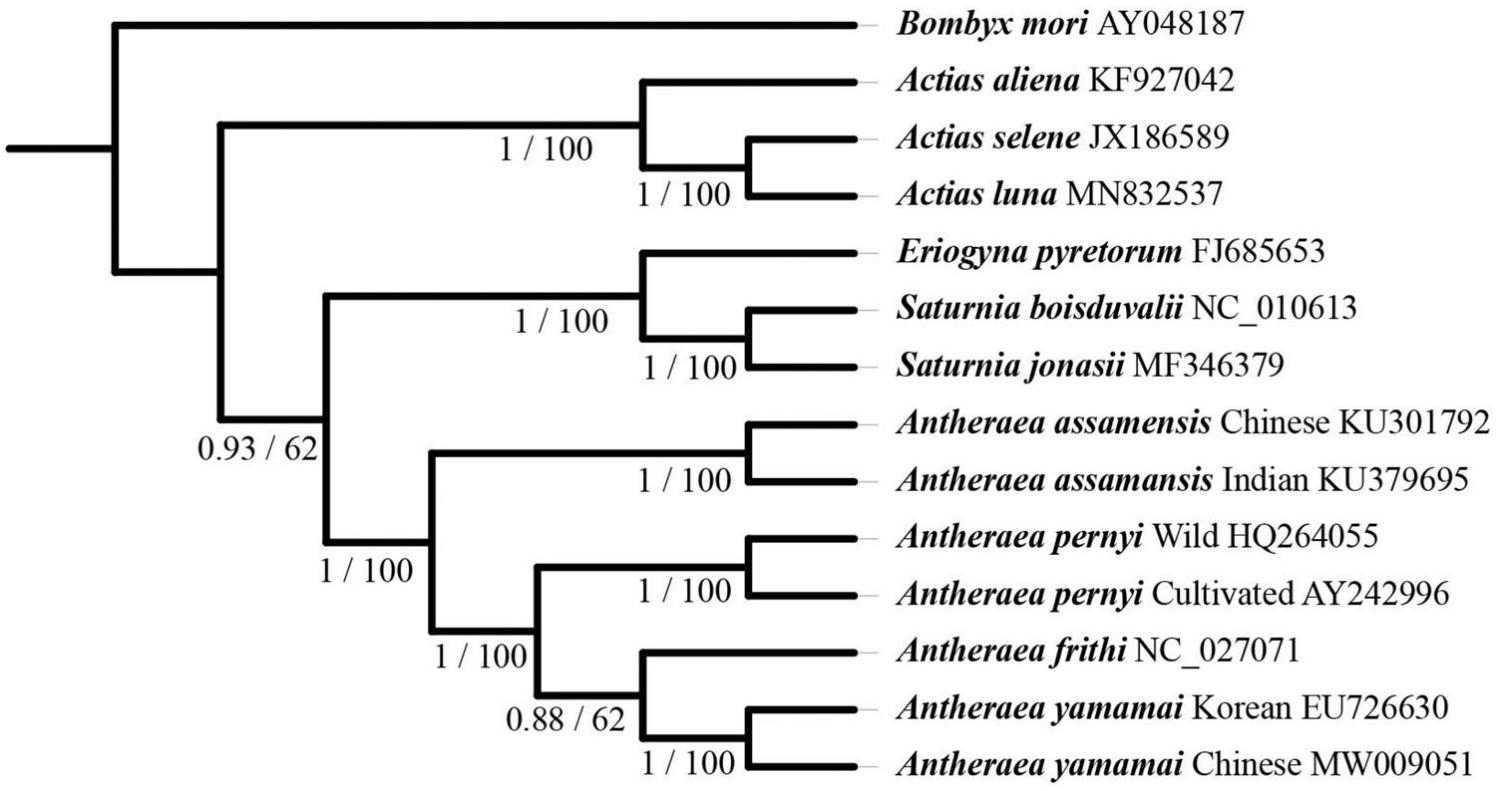
Phylogenetic tree inferred from the whole mitochondrial genome sequence using the maximum-likelihood method and Bayesian inference method with the GTR + F+R3 model. The numbers at each node are Bayesian posterior probabilities (first value) and bootstrap percentages of 1000 replicates (second value). GenBank accession numbers are listed following the scientific name.

## Data Availability

The genome sequence data that support the findings of this study are openly available in GenBank of NCBI at (https://www.ncbi.nlm.nih.gov/) under the accession No. MW009051. The associated BioProject, SRA, and Bio-Sample numbers are PRJNA702206, SRS8270459, and SAMN17928658, respectively.

## References

[CIT0001] Arunkumar KP, Metta M, Nagaraju J. 2006. Molecular phylogeny of silkmoths reveals the origin of domesticated silkmoth, *Bombyx mori* from Chinese *Bombyx mandarina* and paternal inheritance of *Antheraea proylei* mitochondrial DNA. Mol Phylogenet Evol. 40(2):419–427. 16644243 10.1016/j.ympev.2006.02.023

[CIT0002] Hou YB, Wang LZ, Ren SW, Chen LZ. 2010. Current situation and incidence of the wild silkmoth *Antheraea yamamai* from Heilongjiang. Econ Animals Plants. 13(9):10–11. In Chinese.

[CIT0003] Jalili V, Afgan E, Gu Q, Clements D, Blankenberg D, Goecks J, Taylor J, Nekrutenko A. 2020. The Galaxy platform for accessible, reproducible and collaborative biomedical analyses: 2020 update. Nucleic Acids Res. 48(W1):W395–W402. 32479607 10.1093/nar/gkaa434 PMC7319590

[CIT0004] Kim SR, Kim MI, Hong MY, Kim KY, Kang PD, Hwang JS, Han YS, Jin BR, Kim I. 2009. The complete mitogenome sequence of the Japanese oak silkmoth, *Antheraea yamamai* (Lepidoptera: Saturniidae). Mol Biol Rep. 36(7):1871–1880. 18979227 10.1007/s11033-008-9393-2

[CIT0005] Kim SR, Kwak W, Kim H, Caetano-Anolles K, Kim K-Y, Kim S-B, Choi K-H, Kim S-W, Hwang J-S, Kim M, et al. 2018. Genome sequence of the Japanese oak silk moth, *Antheraea yamamai*: the first draft genome in the family Saturniidae. Gigascience. 7(1):1–11. 10.1093/gigascience/gix113 PMC5774507 29186418

[CIT0006] Li XY, Liu YC, Zhang RS, Chen DB, Chen MM, Li YP, Liu YQ, Qin L. 2021. The mitochondrial genome of Qinghuang_1, the first modern improved strain of Chinese oak silkworm, *Antheraea pernyi* (Lepidoptera: Saturniidae). J Insects Food Feed. 7(2):233–243.

[CIT0007] Lu C, Liu YQ, Liao SY, Li B, Xiang ZH, Han H, Wang XG. 2002. Complete sequence determination and analysis of *Bombyx mori* mitochondrial genome. J Agric Biotechnol. 10(2):163–170.

[CIT0008] Nguyen LT, Schmidt HA, von Haeseler A, Minh BQ. 2015. IQ-TREE: a fast and effective stochastic algorithm for estimating maximum-likelihood phylogenies. Mol Biol Evol. 32(1):268–274. 25371430 10.1093/molbev/msu300 PMC4271533

[CIT0009] Ronquist F, Teslenko M, van der Mark P, Ayres DL, Darling A, Hohna S, Larget B, Liu L, Suchard MA, Huelsenbeck JP. 2012. MrBayes 3.2: efficient Bayesian phylogenetic inference and model choice across a large model space. Syst Biol. 61(3):539–542. 22357727 10.1093/sysbio/sys029 PMC3329765

[CIT0010] Shantibala T, Devi KM, Lokeshwari RK, Anju S, Luikham R. 2017. Complete mitochondrial genome of a latent wild oak tasar silkworm, *Antheraea frithi* (Lepidoptera: Saturniidae). Mitochondrial DNA B Resour. 3(1):15–16. 33490481 10.1080/23802359.2017.1413290 PMC7800305

[CIT0011] Singh D, Kabiraj D, Sharma P, Chetia H, Mosahari PV, Neog K, Bora U. 2017. The mitochondrial genome of Muga silkworm (*Antheraea assamensis*) and its comparative analysis with other lepidopteran insects. PLoS One. 12(11):e0188077. 29141006 10.1371/journal.pone.0188077 PMC5687760

[CIT0012] Yue DM, Li SY, Zhang J, Wei Q, Zhao MN, Wang LM. 2017. Analysis on nutrientional content and amino acid composition of *Antheraea yamamai* pupa. Sci Sericult. 43(3):479–485.

[CIT0013] Zhong J, Liu ZH, Yang WK, Zhu F, Dong ZP. 2017. Sequencing and analysis of the complete mitochondrial genome of *Antheraea assama* (Lepidoptera: Saturniidae). Acta Entomol Sinica. 60(8):936–949.

